# Evaluation of Pipe Thickness by Magnetic Hammer Test with a Tunnel Magnetoresistive Sensor

**DOI:** 10.3390/s24051620

**Published:** 2024-03-01

**Authors:** Jun Ito, Yudai Igarashi, Ryota Odagiri, Shigetaka Suzuki, Hiroshi Wagatsuma, Kazuhiro Sugiyama, Mikihiko Oogane

**Affiliations:** 1 Department of Applied Physics, Graduate School of Engineering, Tohoku University, 6-6-05 Aoba-yama, Aoba-ku, Sendai 980-8579, Miyagi, Japan; 2 Fracture and Reliability Research Institute, Graduate School of Engineering, Tohoku University, 6-6-11 Aoba-yama, Aoba-ku, Sendai 980-8579, Miyagi, Japan; 3 Spin Sensing Factory Corporation, Research Center for Rare Metal and Green Innovation, 403 468-1 Aramaki Aza-Aoba, Aoba-ku, Sendai 980-0845, Miyagi, Japan; 4 Center for Science and Innovation in Spintronics (Core Research Cluster) Organization for Advanced Studies, Tohoku University, 2-1-1 Katahira, Aoba-ku, Sendai 980-8577, Miyagi, Japan

**Keywords:** nondestructive testing, magnetic hammer testing, piping, pipeline, tunnel magnetoresistive sensors, tunnel magnetoresistance sensors

## Abstract

A new nondestructive inspection method, the magnetic hammer test (MHT), which uses a compact and highly sensitive tunnel magnetoresistance (TMR) sensor, is proposed. This method complements the magnetic flux leakage method and eliminates the issues of the hammer test. It can therefore detect weak magnetic fields generated by the natural vibration of a pipe with a high signal-to-noise ratio. In this study, several steel pipes with different wall thicknesses were measured using a TMR sensor to demonstrate the superiority of MHT. The results of the measurement show that wall thickness can be evaluated with the accuracy of several tens of microns from the change in the natural vibration frequency of the specimen pipe. The pipes were also inspected underwater using a waterproofed TMR sensor, which demonstrated an accuracy of less than 100 μm. The validity of these results was by simulating the shielding of magnetic fields and vibration of the pipes with the finite element method (FEM) analysis. The proposed noncontact, fast, and accurate method for thickness testing of long-distance pipes will contribute to unmanned, manpower-saving nondestructive testing (NDT) in the future.

## 1. Introduction

### 1.1. Inspection of Pipelines

Steel pipes are an important infrastructure used to transport natural gas, petroleum, hydrogen, and water. As various industries such as oil and gas developed, steel pipes have become longer and more complicated, installed underground and subsea, and some of them are more than 50 years old. Corrosion in piping is caused by acidic gases, hydrogen sulfide, humidity, and pressure [1,2]. If the corrosion is left unchecked for a long time, problems such as leakage accidents and loss of productivity in plants arise [3]. In fact, 20 to 40% of incidents involving piping are due to corrosion [2], and oil, gas, and water companies must continuously inspect their pipeline infrastructure to maintain a safe and stable supply.

The mechanism of corrosion in piping can be categorized into two types: localized corrosion and general corrosion [1,2], both of which cause the inner or outer surface of the pipe to thin. As for pipes supported by pipe racks, localized corrosion occurs when rainwater accumulates in contact with the racks. As for coated pipes, rainwater intrusion can cause “corrosion under insulation” (CUI) or “corrosion under fireproofing” (CUF), both of which cause localized corrosion on the outside surface of the pipe [4].

Various inspection methods, the use of which depends on the use and installation conditions of the piping, have been devised [5]. In Japan, the Japan Petroleum Institute standard (JPI-8S-1-2018) defines methods for inspection of piping [6]. Magnetic flux leakage (MFL) testing has been widely used to detect localized corrosion and flaws [7,8]. MFL detects the leakage of magnetic flux generated by defects in magnetic materials. However, the leakage field is small, and the inspection target (piping) requires magnetization with a permanent magnet or yoke, and that requirement makes the inspection equipment larger. Another method for inspecting piping is the hammer test (HT), in which the pipe is struck directly, and the resulting vibration is analyzed [9,10]. The HT has a relatively low resolution, but it is speedy and simple. However, it is difficult to distinguish sound changes in areas with a lot of background noise, and it has low resolution [11]. In addition, it is difficult to install microphones and accelerometers in the piping to be tested.

The methods to evaluate the thickness of piping include eddy-current testing (ET) and ultrasonic testing (UT), both of which can detect thickness changes in the order of millimeters to microns [12]. ET uses eddy currents to detect scratches on metal surfaces. However, it has the disadvantage of requiring a coil to apply an AC current and a small lift-off of less than 1 mm [5]. UT is widely used for thickness testing because it is easy to measure the thickness with an ultrasonic probe. However, UT requires the sensor to contact the piping [13], and to get close enough to inspect the piping, inspectors must climb up scaffolding erected around the piping every 100 m. Time and money are thus wasted in assembling and removing scaffolding for inspection. At that time, the plant with piping to be inspected must stop operating, and to avoid having to stop operation as much as possible, inspections are conducted once every 10 years. However, the time period between inspections means inspections are infrequent, and within it, the pipeline is at risk of a sudden accident due to failure of the pipeline.

The above-described conventional nondestructive tests (NDTs) are suitable for pipes with localized corrosion. However, pipes underground or underwater do not use pipe racks, so it is necessary to be able to detect general corrosion as well as localized corrosion.

Pipeline inspection gauges (PIGs), in the form of robots equipped with UT, MFL, and ET inspection devices, are used to inspect the inner surface of long underground and underwater pipes efficiently [5,14,15]. Conventional NDT is inefficient for inspecting long-distance piping, so efficient noncontact inspection is needed. However, only a few noncontact, speedy inspection methods, such as capturing the corrosion state of the piping surface by image sensor, are available [5]. This method cannot easily measure the amount of wall thinning, and it requires a light source and a high-sensitivity image sensor.

As described above, to inspect not only rack piping but also underground and subsea piping efficiently and accurately at significantly reduced time and cost, a technology for high-speed, noncontact inspection of piping from the inside surface must be developed. Moreover, the technology must achieve the high safety and high accuracy of NDT without incurring plant shutdowns.

In recent years, to enable inspections even underground and underwater where global positioning system (GPS) cannot be used, autonomous inspections by unmanned aerial vehicles (UAVs) and autonomous underwater vehicles (AUVs) equipped with high-precision inertial-measurement units (IMUs) using micro-electromechanical systems (MEMS) have been developed [15,16]. However, since conventional NDT is inefficient and consumes a lot of power and time, technology that can be adapted to future unmanned/manpower-saving NDT must be developed.

### 1.2. Magnetic Hammer Testing with Tunnel Magnetoresistive Sensor 

NDT must meet five main performance requirements: safety, reliability, ease of use, speed, and running cost [17]. In the case of long pipes, NDT should be able to inspect wall thinning due to corrosion at high speed and low cost, and the inspection device should be compact with low power consumption. Magnetic sensors, whose performance has been remarkably improved in recent years, can meet these requirements. Since steel pipes are ferromagnetic materials, minute corrosion can be easily detected by using a high-performance magnetic sensor to observe the magnetic field that leaks (“leakage magnetic field” hereafter) from steel pipes due to corrosion. 

Magnetic hammer testing (MHT) is very effective for detecting a leakage magnetic field [18]. MHT is an NDT method that detects the size of magnetic material from its natural vibration. In piping, the pressure of the fluid inside constantly generates natural oscillations. The principle of MHT is to detect the slight fluctuation in the spatial magnetic field (mainly geomagnetism) that occurs when the steel material is displaced. Since the oscillation of the magnetic field occurs at the natural frequency of the steel, if it can be detected by a high-sensitivity magnetic sensor, the deterioration state of the steel can be detected from the variation in the frequency. 

MHT is an NDT method that complements MFL and solves the problems of HT. Traditional MFL is a method for detecting local defects based on the strength of magnetic flux leakage, whereas MHT is a way of measuring thickness changes based on frequency. Unlike MFL, it is not affected by the distance between the sensor and the inspection object because it detects structural anomalies from changes in frequency, not magnetic field strength. 

Also, traditional HT is a method in which sounds vibrating at natural frequencies are observed with a microphone, while MHT is a method in which vibration of magnetic fields is observed with a magnetic sensor. When inspecting reinforced concrete, for example, it is difficult to distinguish between them because the microphone measures the sound of both the reinforcing steel and the concrete. Unlike HT, MHT generates no signal from the vibration of objects that do not contain magnetism, such as concrete. In other words, it has the advantage of detecting only signals coming from the steel, which substantially reduces the noise level and may allow inspection with a higher signal-to-noise ratio than conventional HT.

Magnetic oscillations caused by pipe vibrations are so small that a shielded room is conventionally necessary to measure them without the effect of noise. However, the noise can be shielded by the shielding effect of the pipes. Furthermore, magnetization with a permanent magnet or yoke is not required, and the pipe can be inspected anywhere that geomagnetism exists. Pipes can therefore be inspected simply by installing a magnetic sensor inside the pipe in a manner that is much simpler, more efficient, and more accurate than conventional NDT methods. 

MHT requires a magnetic sensor with very low detectivity (high resolution). The detectivity of the magnetic sensor is expressed by the following equation:

(1) $$Detectivity=\frac{S_{v}}{Sensitivity},$$

where $S_{v}$ is noise-amplitude-spectrum density, and the higher the sensitivity of a magnetic sensor, the higher the resolution. The Hall-effect sensor has been widely used as a conventional sensor, but its detectivity is insufficient to detect pT-level magnetic fields [19].

Tunnel magnetoresistive (TMR) sensors have attracted attention in recent years for their high sensitivity and low detectivity. As for the TMR effect, the resistance of an element varies with the relative angle of the two ferromagnets comprising the element. This effect occurs in a magnetic tunnel junction (MTJ), which consists of a thin insulating layer (a tunnel barrier) sandwiched between two ferromagnetic layers. The change in relative resistance of the MTJ is defined as the TMR ratio. Since a TMR ratio of about 20% was observed at room temperature in 1994 [20], the TMR ratio has dramatically improved, reaching as high as 600% today [21]. Along with this improvement in the TMR ratio, the detectivity of TMR sensors has dramatically improved with advances in NDT technology. Combining ET with a TMR sensor enables the detection of scratches of a few millimeters on metal surfaces [22]. In advanced medical applications, TMR sensors can measure magnetocardiography (MCG) signals and nuclear magnetic resonance (NMR) signals [23]. These targets, low-frequency and weak magnetic fields below nT, could not be observed by conventional magnetic sensors but could be observed by TMR sensors with ultra-small detectivity. From the above-described developments, it is clear that the TMR sensor is the most suitable one for MHT in terms of its detectivity properties.

During natural vibration detection with MHT, TMR sensor detectivity contributes significantly to easily achieving a high signal-to-noise ratio by measuring real-time, without averaging over signals. In our previous work, we used a TMR sensor to observe magnetic oscillations in steel plates, and we were able to estimate the millimeter-order thickness of the steel plates from their vibration frequency [18]. In addition, Takata et al. found a proportional relationship between pipe-wall thickness and the vibration frequency of pipes [24]. These results demonstrate that the thickness of pipes made of steel can be easily measured from their natural frequencies. The novelty of this method is that the thickness of pipes can be precisely evaluated from the frequency of the magnetic signal. The TMR sensor, which operates at room temperature, is the only device that can detect minute changes in the natural vibration of pipes at actual infrastructure inspections. Highly sensitive TMR sensors with small device sizes play an important role in inspecting infrastructure with high precision in harsh environments, such as deep water and explosive hazard areas, where it is difficult for inspection workers to approach. For waterproofing, a protective cover can be simply used, but it must be sturdy enough to withstand the water pressure in the sea. To use magnetic sensors in ever harsher environments, the sensor becomes increasingly large, and its spatial resolution becomes worse. However, because TMR sensors are compact and require only a small protective cover, NDT using TMR sensors can maintain high spatial resolution. MFL requires magnetization of the target using a yoke [7]. In contrast, MHT does not need magnetization; it only needs a TMR sensor [18]. Therefore, MHT using TMR sensors requires a smaller device than conventional NDT methods.

Second, TMR sensors have high sensitivity with little power contributing to inspecting pipelines that are at risk of exploding, such as oil and gas. If oil and gas pipes are to be inspected, explosion-proof measures are necessary. For example, H. Vallen et al. are developing a system that allows NDT with acoustic emission (AE) sensors to be used in zones with explosion hazards [25]. Explosion protection requires compliance with national and international standards, with particularly strict limits on voltage and current. The IEC60079 series of the International Electrotechnical Commission (IEC) limits the performance of products in potentially explosive areas (Zone-0) [26]. Within that series, 60079-11 considers electromechanical devices with a maximum rating of 1.5 V, 100 mA, and 25 mW as not likely to be a source of ignition. Current TMR sensors consume less than 25 mW, which will be further lowered in the future [27], allowing TMR sensors to be used safely in potentially explosive areas. Currently, TMR sensors are being made more sensitive, and if they can produce higher output at lower voltages, they will be able to maintain a high signal-to-noise ratio (SNR) even when inspecting piping in hazardous areas.

Another benefit of low power consumption is the small capacity of batteries required to drive infrastructure inspection robots, such as small UAVs and AUVs. In addition, the low voltage means that the risk of MTJ dielectric breakdown is low, and the life of the sensor is long.

In consideration of the above-described improvements, it is clear that MHT using a TMR sensor is a very useful NDT method for long-distance piping, and the TMR sensor is also consistent with future automated and manpower-saving nondestructive inspection. In addition, if TMR sensors are made waterproof and explosion-proof, their utility could be exponentially expanded, and NDT technology could be dramatically advanced.

As a first step to investigate the applicability of MHT to inspection of piping, in this study, we attempted to measure the weak magnetic field associated with the natural vibration of piping by striking it with a hammer. As the next step, namely, to clarify the applicability of using MHT underwater, we attempted to inspect underwater piping by using a waterproof TMR sensor. Additionally, we used structural analysis to evaluate the vibration characteristics of the piping. Finally, we used structural–magnetic combined analysis to evaluate the attenuation of the magnetic field due to the shielding effect of the piping and the variation in the magnetic field in the piping.

## 2. Experimental Setup

### 2.1. Test Specimens and Experimental Setup

The experimental setup for investigating the applicability of MHT to inspection of piping was located outside a magnetically shielded room exposed to a geomagnetic field. This investigation targeted carbon steel widely used for oil and gas piping or water pipes, namely, Japanese Industrial Standards (JIS) STPG370 (370 N/mm^2^ grade steel) [28], which is characterized by its sufficient toughness for use as gas and oil piping (Young’s modulus, (E) 205 GPa; density (ρ), 7850 kg/m^3^; and Poisson’s ratio (ν), 0.3) [24]. The reference specimen is a pipe with 1000 mm length (L), 60 mm diameter (D), and 5.66 mm thickness (h).

**Figure 1:**
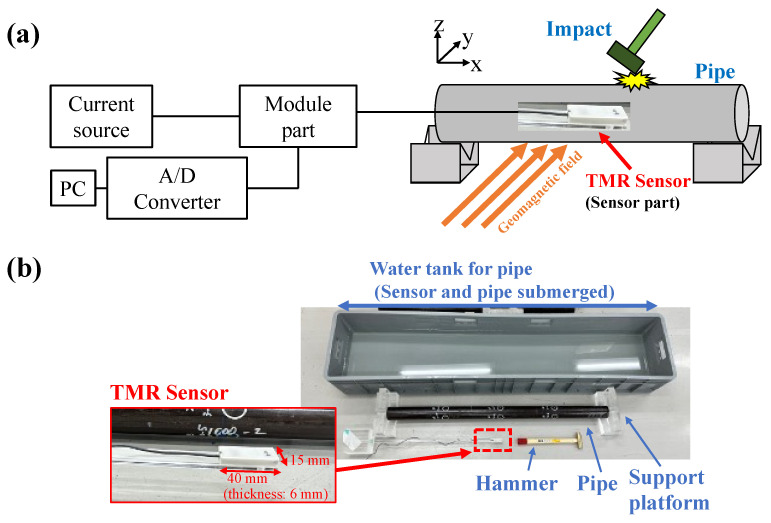
(**a**) Schematic diagram of the experimental setup of the magnetic hammer testing (MHT) inspection system and (**b**) photograph of the experimental setup.

The configuration of the experimental MHT inspection system is shown in Figure 1. A brass hammer is used to strike the center of the top surface of the test pipe (“specimen” hereafter) to generate the natural vibration of the pipe. Since different inspectors will naturally apply different striking forces, their hammer strikes are expected to produce variations in the spectral amplitude of the natural vibration of the pipe. However, frequency is the only important parameter in MHT, so differences in striking force are not a significant issue. The specimen is vibrated under rigid-support (SR-SR) boundary conditions maintained by a support platform, so neither end of the specimen is displaced. The acrylic-made support platform has a V-shaped groove that can support piping of any diameter. The pipe is placed in the direction orthogonal to the geomagnetic field. 

The TMR sensor is placed in the center of the pipe and oriented parallel to the long axis of the pipe. The dimensions of the TMR sensor are 40 mm for the long side, 15 mm for the short side, and 6 mm for the thickness. The TMR sensor is waterproofed to prevent water from adhering to it as shown in Figure 1b. A 3D printer was used to fabricate a protective cover for the sensing part, which was then solidified with silicon resin, and a vacuum pump was used to remove air bubbles.

To evaluate the practicality of the MHT inspection system underwater, an underwater experiment under the same SR-SR boundary conditions, as described above, was attempted. In the underwater experiment, the tank was filled with water, and the specimen (housing the TMR sensor and supported by the same platform used in the in-air test setup) was submerged in the water. The equipments were not waterproof except for the TMR sensor. A hammer was also submerged in the water and struck the piping underwater. The signal generated by the blows was acquired by the waterproofed sensor. The experimental setup for signal transmission is the same as in the aboveground case.

### 2.2. Sensor Performance

**Figure 2:**
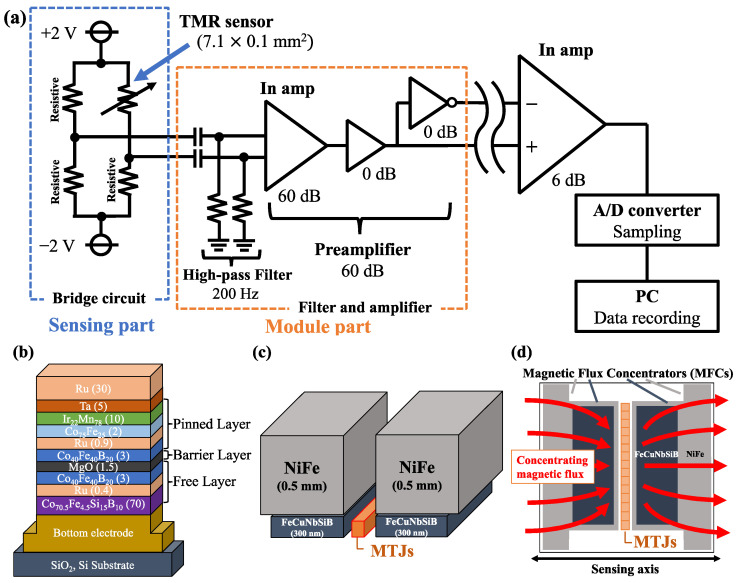
(**a**) Schematic diagram of the MHT measurement system using TMR sensor (In amp: instrumentation amplifier), (**b**) the magnetic tunnel junction (MTJ) device, (**c**) magnetic flux concentrators (MFCs), and (**d**) top view of (**c**).

The TMR sensor and its electrical circuit are shown schematically in Figure 2. To read out the minute resistance of an MTJ device with high accuracy, devices such as bridge circuits and A/D converters are needed. As shown in Figure 2a, the TMR sensor consists of a sensing part and a module part. The sensing part consists of a bridge circuit including a TMR sensor. A voltage of ±2.0 V is supplied to the bridge circuit, which outputs a differential voltage. 

The TMR sensor consists of 74 MTJ devices as shown in Figure 2d. The area of the arrayed MTJ device is $7.1\times 0.1\;{mm}^{2}$. MTJ devices were deposited by using an ultra-high-vacuum magnetron sputtering system. The stacking structure was Substrate Si, SiO_2_/bottom electrode/CoFeSiB (70)/Ru (0.4)/CoFeB (3)/MgO (1.5)/CoFeB (3)/Ru (0.9)/CoFe (2)/IrMn (10)/Ta (5)/Ru (30) (the thicknesses shown in parentheses are in nanometers) as shown in Figure 2b. In the pinned layer at the top of the MTJ, a spin-valve structure with CoFeB and IrMn is used to enable positive and negative readings of the magnetic field. To fabricate a thin barrier layer, a Mg layer with a thickness of 0.1 nm was deposited by in situ natural oxidization, and the deposition was repeated several times to achieve a MgO layer with a thickness of 0.7 nm. In the free layer at the bottom of the MTJ, a synthetic structure of CoFeSiB and CoFeB reflects excellent soft magnetic properties due to weak magnetic field coupling. 

The signal-to-noise ratio (SNR) of the TMR sensor is improved in proportion to the square root of the number of MTJ devices in series. To reduce 1/*f* noise, 74 MTJ devices are connected in series. To further improve SNR, two types of magnetic flux concentrators (MFCs) were installed on both sides of the MTJ arrays as shown in Figure 2c,d. First, a thin MFC film made of FeCuNbSiB (300 nm thick) was deposited in the immediate vicinity of the MTJ arrays, and a bulk MFC (0.5 mm thick) made of NiFe was installed. The bulk MFC is T-shaped and its size is 15 mm in the direction of the sensing axis and 10 mm in the direction parallel to the MTJ arrays. The combination of these materials and structures dramatically increased the sensitivity of the TMR sensor and achieved a detectivity of 50 fT/√Hz@1 kHz [23]. The TMR sensor was manufactured by the Spin Sensing Factory Corporation (Sendai, Japan).

To prevent water from adhering to the module part, it is placed at a certain distance from the sensor part, but the greater the distance, the more noise is likely to enter the circuit. To eliminate the noise, therefore, the output of the bridge circuit in the sensing part is received by the differential amplifier in the module part as shown in Figure 2a.

To achieve a high SNR through further noise reduction and signal amplification, a 200 Hz high-pass filter and a 60 dB amplifier are installed in the module part. The amplified and noise-eliminated output signal is converted to a digital signal by an A/D converter (PowerLab 16/35, AD Instruments, Dunedin, New Zealand) with a sampling frequency set at 40 kHz. The digital signal is recorded by Labchart8 (AD Instruments, Dunedin, New Zealand) installed on a PC.

### 2.3. Sensitivity Calibration

**Figure 3:**
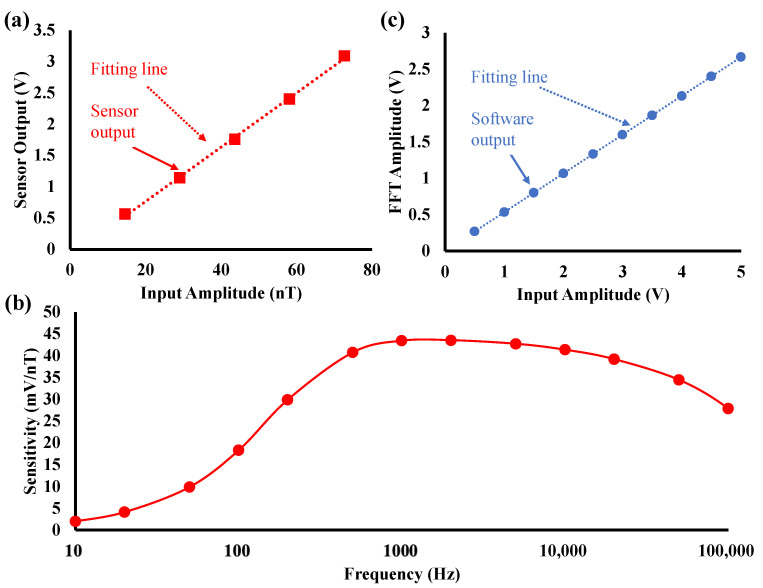
(**a**) Results of sensitivity calibration of TMR sensor at 1 kHz, (**b**) results of the frequency response of the sensitivity of TMR sensor, and (**c**) results of calibration of A/D converter (PowerLab, AD Instruments) and software (LabChart, AD Instruments).

The sensitivity of the TMR sensor was calculated by applying an AC magnetic field via a solenoid coil, which is connected to a function generator via a resistor to generate a magnetic field from 10 Hz to 100 kHz. The output of the coil is flat up to 200 kHz. The voltage required by the function generator for the coil to generate a unit magnetic field is 0.14 $V_{p-p}/\mu T$ from 10 Hz to 200 kHz. The input amplitude was controlled by the function generator, and the AC output generated by the TMR sensor was recorded. The relationship between the input magnetic field and the output of the sensor is shown in Figure 3a. The sensitivity of the TMR sensor is 43.39 mV/nT@1 kHz. The frequency response of sensitivity of the TMR sensor is shown in Figure 3b. The small sensitivity below 200 Hz is due to the high-pass filter. The sensitivity drop above 1 kHz can be attributed to the effect of amplifiers in the module part.

The signal output from the TMR sensor is transformed into a spectrum by using a fast Fourier transform (FFT) given by LabChart8 software (version 8.1.20). The relationship between the PowerLab input signal and the FFT result (FFT amplitude) obtained using LabChart is shown in Figure 3c. It was obtained by connecting a function generator to PowerLab and inputting AC voltage to the function generator. The result is 0.5 FFT amplitude/V. 

### 2.4. Vibration Properties of Pipe

One target of this study to investigate the applicability of MHT to inspection of piping is the natural vibration of a magnetic material (steel pipe), and the vibration of the magnetic field is based on the vibration behavior of an elastic body. Therefore, the theory of the natural vibration of a cylindrical shell (steel pipe) is explained hereafter. 

The axial vibration of a cylindrical shell is described first. It can be explained using the same theory as used for beams, if they are supposed to be hollow beams, the natural frequency becomes

(2) $$f=\frac{\lambda^{2}}{2\pi L^{2}}\sqrt{\frac{EI}{\rho A}},$$

where *E* is Young’s modulus (Pa), *I* is the second moment of area, and *EI* is the flexural rigidity of the pipe [29]. Note that ρ is the volume density of the pipe (kg/m^3^), *A* is the cross-sectional area of the pipe (m^2^), *L* is the length of the pipe (m), and λ is the boundary conditions, which are 4.730, 7.853, and 10.996 for the first, second, and third modes under rigid support (SR-SR), respectively. Thus, the axial natural frequency is mainly determined by the length of the pipe.

Next, the circumferential vibration of a cylindrical shell is explained. The classical theory of cylindrical shells was devised by Lord Rayleigh in the form of the following equation [30]:

(3) $$\omega^{2}=\frac{h^{2}}{12R^{2}}\frac{E}{\rho R^{2}\left(1-\nu^{2}\right)}\frac{n^{2}{\left(n^{2}-1\right)}^{2}}{n^{2}+1},$$

where ν is Poisson’s ratio, h is the wall thickness of the shell (m), n is the circumferential order, namely, an integer greater than or equal to the second order (when the first order is ignored), and *R* is the center radius of the shell (steel pipe) expressed as follows:

(4) $$R=\frac{D/2+d/2}{2},$$

where *D* is the outer diameter and *d* is the inner diameter of the pipe. Equation (3) is based on the assumption of infinitely long pipes and no expansion or contraction in the plane. About 100 years after Rayleigh, Koga presented a unified theory based on various classical theories after Rayleigh’s in the form of the following equation [31]:

(5) $$\omega^{2}=\frac{h^{2}}{12R^{2}}\frac{E}{\rho R^{2}\left(1-\nu^{2}\right)}\frac{n^{2}{\left(n^{2}-1\right)}^{2}}{n^{2}+1}\left\{1+\frac{\alpha \xi^{4}}{{\left(n^{2}-1\right)}^{2}}\right\},$$

(6) $$\alpha =\frac{12\left(1-\nu^{2}\right)R^{2}}{h^{2}},$$

where ξ is the boundary condition, and if the ratio of length to radius of the pipe is l=(L/2)/R, the following equations are obtained when the boundary condition is SR-SR:

(7) cosh2nξlcos2nξl−1=0,

(8) $$\xi =\frac{\lambda }{2nl}=\frac{4.730}{2nl},$$

The natural frequencies of the circumferential vibration modes are therefore expressed as follows:

(9) $$f=\frac{h}{2\pi R^{2}}\sqrt{\frac{E}{12\rho \left(1-\nu^{2}\right)}}\sqrt{\frac{{\left(n^{2}-1\right)}^{2}+\alpha {\left(R\lambda /nL\right)}^{4}}{1+1/n^{2}}}.$$

Equation (9) is highly accurate for cylindrical shells with ${\left(1-\nu^{2}\right)}^{1/2}{\left(L/R\right)}^{2}{\left(R/h\right)}^{2}\geq 200$ [32]. Circumferential frequency is mainly determined by pipe thickness, and these parameters are proportional. In the case of pipes longer than 3 m, pipe length does not depend on the natural frequency, which is the same as that of a circular ring [24]. In addition, circumferential vibration is excited by turbulence and is especially pronounced when the wall is thinner and less stiff [33]. Because circumferential vibration depends on pipe thickness, the best target for pipe-thickness inspection by MHT is circumferential vibration.

Finally, the general theory for pipe thickness is explained. Ideal piping has uniform pipe thickness; but in actual cases, pipe thickness is not uniform due to process variations. Therefore, the average pipe thickness $\bar{h}$ is given as follows [34]:

(10) $$\bar{h}=\frac{\int_{-L/2}^{L/2}\int_{0}^{2\pi }h\left(z,\theta \right)Rd\theta dz}{\int_{-L/2}^{L/2}\int_{0}^{2\pi }Rd\theta dz}.$$

In this study, the thickness of the reference pipe was measured at certain points along the pipe’s length by experts (using ultrasonic testing by ENEOS), and average pipe thickness was calculated from the measurements. Therefore, the pipe is treated as having uniform thickness with average pipe thickness $\bar{h}$.

### 2.5. Magnetic Properties of Pipe

Another target of this study is to measure the leakage magnetic field of the pipe. To describe the magnetic properties of the pipe, the pipe is treated as a cylindrical magnetic material. First, the magnetic field generated by the pipe is obtained from the following equations [8,35]:

(11) $$V\left(r\right)=V_{a}\left(r\right)+V_{d}\left(r\right),$$

(12) $$V_{d}\left(r\right)=\frac{1}{4\pi }\int_{v}\frac{\rho_{m}}{\left|r\right|}dv+\frac{1}{4\pi }\int_{s}\frac{\sigma_{m}}{\left|r\right|}ds,$$

where V is a scalar potential defined by $B=-\mu_{0}\nabla V$; $V_{a}$ and $V_{d}$ are the potentials due to the exterior and interior of the magnetic material, respectively; $\rho_{m}$ is the magnetic charge inside the magnetic material, $\sigma_{m}$ is the magnetic charge induced on the surface, and both are defined by magnetization *M*, where $\rho_{m}=-\nabla \cdot M$ and $\sigma_{m}=n\cdot M$; n is a vector orthogonal to the surface; and r is a vector from the magnetic material to an arbitrary position in space. From Equation (11), the magnetic field created by the pipe is given as follows:

(13) $$B\left(r\right)=B_{a}\left(r\right)+B_{d}\left(r\right),$$

(14) $$B_{d}\left(r\right)=\frac{\mu }{4\pi }\int_{v}\frac{\rho_{m}}{{\left|r\right|}^{3}}rdv+\frac{\mu }{4\pi }\int_{v}\frac{\sigma_{m}}{{\left|r\right|}^{3}}rdv,$$

where μ is magnetic permeability, defined by free-space permeability $\mu_{0}$ and relative permeability $\mu_{r}$, where $\mu =\mu_{0}\mu_{r}$. Note that $\mu_{r}$ of air is 1, while that of steel is about 100 to 1000. $B_{a}$ is the external magnetic field, and the geomagnetic field is about 50 μT. Therefore, the magnitude of the magnetic field created by the piping is determined by the distance from the piping. In other words, the natural frequency can be detected from the oscillation of the magnetic field because the distance to the magnetic sensor changes from moment to moment as the piping vibrates. In this study, the static magnetic field was calculated by the finite element method (FEM).

The shielding properties of pipes are discussed next. The mechanisms of shielding associated with pipes can be classified into two categories depending on the frequency of the input magnetic field [36,37]. Shielding in the case of static or low-frequency magnetic fields can be explained by the flux-shunting mechanism, while that in the case of high-frequency fields can be explained by eddy-current cancellation. Flux-shunting allows the magnetic flux to pass through the pipe, while avoiding the inside of the cylinder, so very little flux remains inside the pipe. Geomagnetic shielding and environmental-noise shielding are due to flux-shunting. 

Attenuation of the magnetic field can be explained by using the shielding factor, which is determined by the dimensions of the pipe and magnetic permeability $\mu_{r}$. When the pipe is of finite length, the axial components of the shielding factor are respectively as follows [35,38]:

(15) $$\lambda_{a}=\frac{1}{1+\mu_{r}\frac{4h}{D}N_{a}},$$

(16) $$N_{a}=\frac{1}{T^{2}-1}\left[\frac{T}{\sqrt{T^{2}-1}}ln\left(T+\sqrt{T^{2}-1}\right)-1\right],$$

where *T* = *L*/*D*. From this factor, the radial shielding factor is obtained as follows:

(17) $$\lambda_{r}=\frac{1}{1+\mu_{r}\frac{2h}{D}\left[1+T^{-2}\right]N_{a}},$$

(18) $$N_{r}=\frac{1}{2}\left(1-N_{a}\right)\frac{4}{\pi }.$$

As shown in these equations, the shielding factor is determined by *L*/*D* and *h*/*D*. In this study, the short pipe (1000-mm length) is placed in the geomagnetic field, and the magnetic field in the pipe is expected to be smaller than outside due to the shielding effect of the pipe walls. 

## 3. Results

### 3.1. Time Waveform and Spectrum of MHT Signal

**Figure 4:**
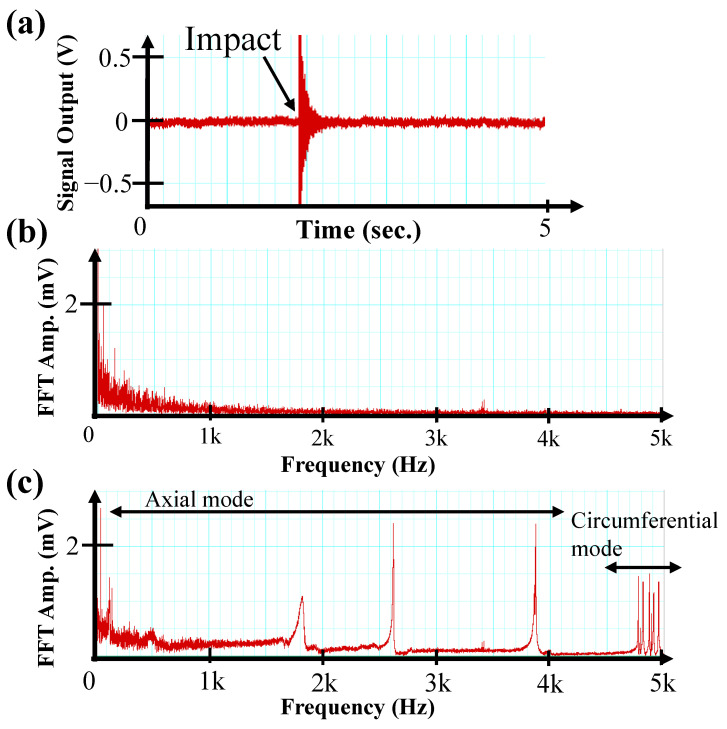
(**a**) Time profile of TMR output, (**b**) spectrum of background noise (before impact), and (**c**) spectrum of the signal from the pipe vibration due to impact of the hammer.

The results of the MHT measurement of the reference pipe by using the TMR sensor are shown in Figure 4a. The time to operate the TMR sensor was 5 s, and the pipe was struck only once by the brass hammer. Except for the impact of the hammer on the pipe, the signal output from the sensor contains only background noise generated by the measurement environment and experimental setup. It increased significantly due to the impact of the brass hammer (at around 3 s as shown in Figure 4a).

To calculate the natural frequencies of vibration of the pipe, the resulting voltage–time waveforms were converted to amplitude vs. frequency spectra by using an FFT. The spectrum of the background noise, which appeared mostly in the low-frequency range, is shown in Figure 4b. The spectrum of the MHT signal with an FFT amplitude of about 2 mV is shown in Figure 4c. The peak of the MHT signal is apparently larger than the background noise in the high-frequency range.

As can be seen from the spectra in Figure 4, the peaks appear discretely in the region below 4 kHz, while they appear in groupings in the region above 4 kHz. This result is explained by the difference in the characteristics of axial and circumferential vibration modes, and it is in good agreement with the relationships shown by Equations (2) and (9). In the case of the reference piping, it was found that axial vibration modes appeared in the region below 4 kHz and circumferential vibration modes appeared in the region above 4 kHz.

The above-described findings demonstrate that both axial and circumferential vibrations of the piping could be observed by MHT using a TMR sensor. Since signals originating from low-frequency environmental noise or axial vibration can be easily removed by a high-pass filter, a highly accurate evaluation of pipe thickness by observation of circumferential vibration is possible.

Even though the MHT experiment was conducted outside a magnetically shielded room, the environmental noise peak hardly appeared compared to the natural-frequency peak. This result is due to the magnetic shielding effect of the piping, indicating that MHT can be performed in a low-noise environment by installing the sensor inside the pipe. The shielding effect of the pipe is explained in Section 4.2.

### 3.2. Dependence of Natural Frequency on Pipe Thickness

To investigate the relationship between pipe thickness and frequency of circumferential vibration, several steel pipes with thicknesses of 5.66, 5.07, 4.82, and 4.57 mm and constant length were prepared. The 5.66 mm pipe was the reference pipe, and the other three pipes were corrosion simulation pipes, which were shaved from the 5.77 mm pipe. In addition, none of the pipes were magnetized, and they were in clean condition with no flaws.

The setup for this experiment is the same as in Figure 1. A brass hammer is used to strike the center of the top surface of four specimens. The pipes are vibrated under SR-SR boundary conditions maintained by a support platform. The TMR sensor is placed in the center of the pipes and oriented parallel to the long axis of the pipes. The pipes are placed in the direction orthogonal to the geomagnetic field. 

Since the amplitude of circumferential vibration varied with different striking forces, five tests were conducted, and the average spectrum of circumferential vibration was calculated from the following equation:

(19) $$S_{Ave}\left(f\right)=\frac{\sum_{n}S_{n}\left(f\right)}{5},$$

where $S_{Ave}\left(f\right)$ is the average spectra at *f* (Hz), and $S_{n}\left(f\right)$ is the value of the spectrum at *f* (Hz) obtained at the *n*-th test.

After five tests for each of the four pipes, the sensor, specimens, and the support platform were submerged in a tank filled with water for the underwater experiment. The boundary conditions were the same SR-SR boundary conditions. The hammer was also submerged in water to strike the pipes. The position and orientation of the TMR sensor are the same as in the experiment for the aboveground case. Five tests were also conducted in water on each of the four pipes.

In addition, to analyze the frequency-response spectrum, a Lorenz function was fitted to the average spectrum of circumferential vibration by using Python [39]. The Lorenz function is given as follows:

(20) $$f\left(x;A,\mu ,\sigma \right)=\frac{A}{\pi }\left[\frac{\sigma }{{\left(x-\mu \right)}^{2}+\sigma^{2}}\right],$$

where *A* is the amplitude, *μ* is the center, and *σ* is the sigma. Note that *A*, *μ*, and *σ* are parameters of the average spectrum. In the Lorenz function, 2*σ* means the full width at half maximum (FWHM). The function was fitted to the power spectrum to minimize the sum of squares of the residuals.

**Figure 5:**
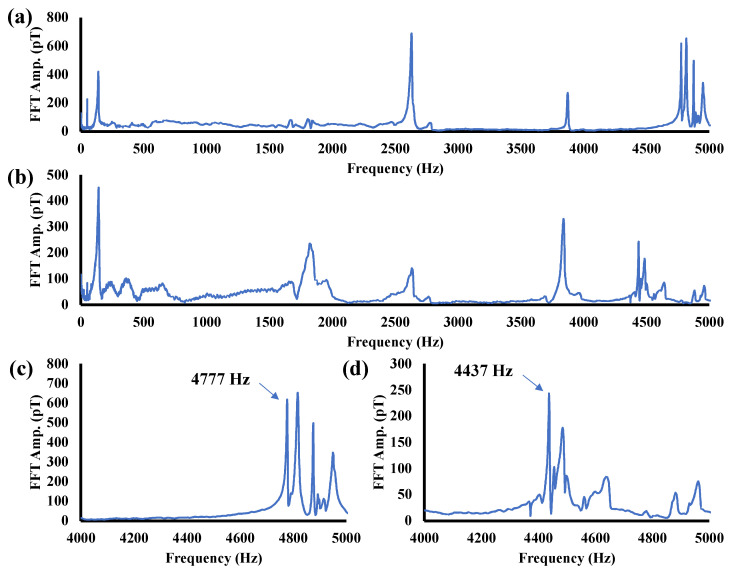
(**a**) Spectrum for 5.66 mm thick pipe and (**b**) 5.07 mm thick pipe; (**c**) enlarged view of (**a**) and (**d**) enlarged view of (**b**).

The average spectra obtained by MHT with the 5.66 and 5.07 mm thick pipes are shown in Figure 5a,b. Comparing the two figures reveals that several peaks observed below 4 kHz are at the same position regardless of thickness. These peaks correspond to the axial natural vibration of the pipe. On the other hand, the peaks observed above 4 kHz show a systematic variation with respect to thickness. As shown in Figure 5c, the averaged spectrum for the reference pipe shows a clear peak of 617 pT originating from the natural frequency of 4777 Hz. A peak of 237 pT originating from the natural frequency of 4437 Hz of the 5.07 mm thick pipe is shown in Figure 5d. 

**Figure 6:**
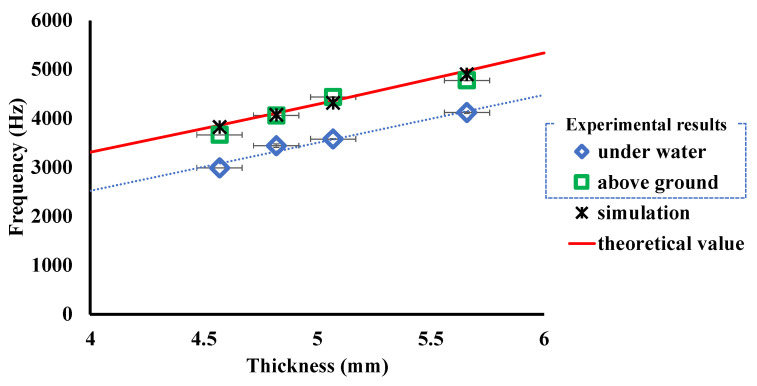
Relationship between natural frequency and thickness of pipe.

These results suggest the possibility of accurately discriminating pipeline thickness differences from sub-nT magnetic fields originating from natural vibration. The dependence of circumferential natural frequency on pipe thickness was evaluated by using pipe specimens with four different thicknesses. The relationship between pipe thickness and the natural frequency evaluated using experimental values, theory, and FEM analysis is shown in Figure 6. The red lines are theoretical values obtained from Equation (9). The black markers are values obtained from finite-element simulations, the settings of which are given in Section 4.1. Both simulation results and theoretical values were computed with the boundary condition SR-SR, and both results agreed well.

The blue markers are the results of the underwater experiment. They differ from the theoretical, simulated, and experimental results aboveground. The difference that occurred underwater may be due to the fact that the boundary conditions of the submerged pipes changed due to the buoyancy of water on the pipes. However, a linear relationship between natural frequency and pipe thickness was obtained even underwater. This suggests that the relationship between natural frequency and thickness of piping is linear, independent of boundary conditions. This key finding can be explained by the theory of natural vibrations. From Equation (9), the following relationship holds

(21) $$\frac{df}{dh}=\frac{f}{h}.$$

Equation (21) does not include any parameters regarding boundary conditions, which suggests that natural frequency and the thickness of the pipeline have a linear relationship regardless of how the specimen is clamped.

From the above-described results, it is clear that MHT with a TMR sensor can be used to estimate pipe thickness from the difference in natural frequencies regardless of the boundary conditions. In the case of pipes longer than 3 m, the natural frequency is not dependent on pipe length [24], so the pipe thickness may be estimated with even higher accuracy.

### 3.3. Evaluation of Resolution of Pipe-Thickness Measurement 

The resolution of the pipe-thickness measurement was calculated from experimental results shown in Figure 5 and Figure 6. The change in natural frequency per unit thickness (“sensitivity” hereafter) can be defined from the linear relationship between pipe thickness and natural frequency and is expressed as follows:

(22) $$Sensitivity=\frac{df}{dh}\left(Hz/mm\right).$$

The higher the sensitivity, the easier it is to observe changes in thickness. In this study, the sensitivities were 990.3 ± 155.6 Hz/mm aboveground and 979.9 ± 108.6 Hz/mm underwater. As mentioned earlier, the difference in sensitivity was caused by the difference in natural frequencies due to changes in boundary conditions.

Measurement accuracy and resolution are given as follows:

(23) $$Accuracy=FWHM\;\left(Hz\right),$$

(24) $$Resolution=\frac{FWHM}{Sensitivity}\left(mm\right).$$

Accuracy for the reference pipe (5.66 mm thick) was 4 Hz aboveground and 23 Hz underwater. From Equation (24), the minimum resolution for the reference pipe was calculated to be 4.4 ± 0.7 μm aboveground and 23.6 ± 2.6 μm underwater. 

The pipe wall thickness can be estimated using Equation (21). Since the uncertainty of the boundary conditions causes large errors in the pipe thickness (*h*), the relative value of the pipe thickness (Δ*h*) that is less affected by boundary conditions has validity. The relative value of the pipe thickness can be estimated from the difference in natural frequencies using the following equation:

(25) $$∆h=∆f/\frac{df}{dh}\;\left(mm\right).$$

From Equation (25) and Table 1, the change in natural frequency determines the wall thickness of the pipe. When evaluated using the sensitivity (990.3 ± 155.6 Hz/mm) obtained for four specimens, including a 5.66 mm pipe, the error was larger than 100 μm. On the other hand, using the sensitivity (1544.3 ± 16.7 Hz/mm) acquired for three pipes (5.07 mm, 4.82 mm, and 4.57 mm), we confirmed that it could indeed be estimated with an accuracy of less than 10 μm. The large error in the 5.66 mm pipe is assumed to be due to the ±100 μm error in the ultrasonic measurement test (UT). 

### 3.4. Discussion of the Experimental Results

In this study, a thickness resolution of several tens of micrometers aboveground and less than 100 μm underwater was achieved. These values are much higher than the resolution of a typical UT. In addition, even if the average pipe thickness varies by a few micrometers, it will be detectable by the highly accurate MHT.

According to Equation (20), amplitude is inversely proportional to *σ*, and FHWM is proportional to *σ*. If the specimen has no flaws or defects, it will vibrate ideally, so the damping time tends to be long, and the FHWM tends to be narrow. In this study, the damping time of the vibration of the pipe with reduced wall thickness was shorter and the FWHM of the vibration was wider. This finding indicates that the degree of corrosion determines the resolution of the pipe-thickness measurement. 

As expected, measurement accuracy was worse underwater than aboveground, due to shorter damping time. The fact that higher accuracy than UT was achieved despite noncontact measurements has a high significance. The achievement of sub-millimeter resolution in both aboveground and underwater experiments contributes to the greater practicality of MHT.

The high resolution achieved in this study is due to the high sensitivity defined by the natural frequency of the piping and the small detectivity of the TMR sensor. In the case of steel plates, the sensitivity is 119 Hz/mm and the resolution is 0.3 mm [18]. Therefore, MHT may be able to achieve high resolution not only with steel plates and pipes but also with other steel materials, such as circular steel plates like manhole covers.

However, environmental noise may increase when used in the real world, and the results of this experiment performed in a low-noise environment are considered near-limitation. The challenge to achieve high thickness resolution in the real world is to further improve TMR sensor detectivity. According to Equation (1), the detectivity can be lowered by improving the sensor noise level and sensor sensitivity. Sensor noise mainly requires improvement of the barrier layer of the MTJ device. In addition, for sensitivity, optimization of the free layer of the MTJ device, film FC, and bulk FC of the sensor is important.

While MHT is a highly accurate method for evaluating the average wall thickness of pipes, it may be disadvantageous for obtaining dimensions on local defects like small holes. According to Equations (10) and (25), when a circular hole of 5 mm diameter and 4.56 mm depth is made in the reference pipe, the natural frequency change is only 0.2 Hz. This is smaller than the accuracy of this experiment, so small holes cannot be measured with the MHT. To overcome this disadvantage, it is effective to use MFL in combination with MHT, which can observe hole diameter, hole depth, and hole location. By using MFL in combination with MHT, it would be possible to simultaneously observe local defects and average wall thinning in the pipe with a single sensor.

## 4. Simulation Results Using FEM

### 4.1. Modeling and Structural Analysis

The oscillating magnetic field generated by the vibration of the pipe was verified using the finite element method (FEM). We first modeled the pipe and air according to the experimental setup and then conducted both structural and magnetic-field analyses of the pipe’s natural vibrations and magnetic field. Note that we used ANSYS (Canonsburg, PA, USA) as our simulation software.

The model was created with the settings shown in Table 2 for structural and magnetic analysis. The settings were based on the dimensions and material of the reference specimen (5.66 mm thick). To investigate the magnetic flux distribution around the steel pipe, an air model surrounding the pipe was also created.

**Figure 7:**
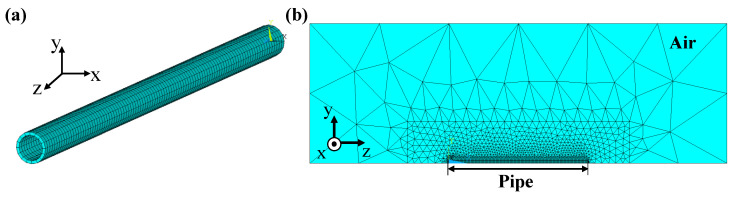
(**a**) Modeling of a steel pipe and creating a ring on the x–y plane and extending it along the z-axis and (**b**) cross-section of a 1/4 model of a pipe and air within it. To create a full model, the 1/4 model is mirror copied in the x–z and x–y planes.

The results of meshing the model are shown in Figure 7. The circumferential direction was meshed into 16 divisions, and the axial direction was meshed into 100 divisions in 1 cm increments. The air inside the pipe was finely meshed with 100 divisions along its length to calculate the magnetic field accurately, while the air outside was meshed roughly to reduce the amount of calculation. The pipe and the air inside it consist of fine hexahedrons, while the air outside consists of rough tetrahedrons, both generated using element type SOLID186.

The modal frequencies and shapes of the axial and circumferential vibration modes were calculated by modal analysis. To extract the several modes of pipes, the mode superposition method was used. In this analysis, only the pipe model was selected to calculate the modal frequencies. 

The governing equation used in the modal analysis is given as follows:

(26) $$\left[M\right]\left\{\ddot{u}\right\}+\left[K\right]\left\{u\right\}=0$$

where $\left[M\right]$ is the mass matrix, $\left\{u\right\}$ is the displacement vector, $\left[K\right]$ is the stiffness matrix. When u(t)={A}cosωt and {A}≠0, the natural frequency is obtained from the following equation:

(27) $$\det\left(\left[K\right]-\omega^{2}\left[M\right]\right)=0$$

The boundary condition was set to zero displacement at both ends of the pipe in the length direction. In addition, external forces and damping were not considered.

**Figure 8:**
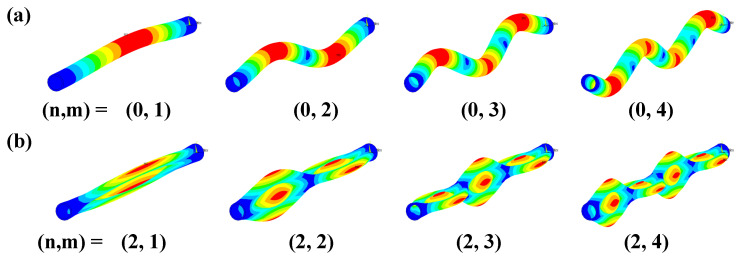
Results of modal analysis with reference sample: (**a**) n = 0 and (**b**) n = 2. Areas of large displacement are shown in red, and areas of small displacement are shown in blue.

The axial and circumferential vibration modes are denoted by m and n, respectively, and the vibration-mode shapes are shown in Figure 8. For axial-only vibration (n = 0), the calculated vibration-mode shapes from m = 1 to 4 are shown in Figure 8a. For n = 2 vibration, the calculated vibration-mode shapes from m = 1 to 4 are shown in Figure 8b. For axial only (n = 0) vibration, the calculated vibration-mode shapes are similar to those of a beam. As for axial behavior, the vibration-mode shapes become more complex as the order of m increases, and the simplest shape occurs when (n, m) = (2, 1).

**Figure 9:**
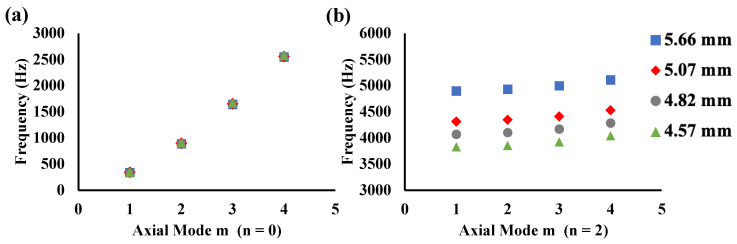
Natural frequencies calculated using modal analysis with all samples: (**a**) n = 0 and (**b**) n = 2.

The relationship between the order of axial mode and natural frequency is shown in Figure 9. Change in natural frequency without circumferential vibration (n = 0) and without dependence of axial vibration on pipe thickness is shown in Figure 9a. On the contrary, as shown in Figure 9b, for n = 2 vibration, the natural frequency is determined by pipe thickness; pipe thickness and natural frequency are not proportional even for modes with m > 3.

From the results of the above-described simulations, pipes with larger diameters have lower natural frequencies. The detectivity of the TMR sensor deteriorates in the low-frequency band, and that deterioration may adversely affect measurement accuracy in the case of thick pipes. Therefore, if we choose a mode with higher order m or n, which is the region of high detectivity of TMR sensors, we will even be able to inspect thick pipes with a high SNR.

### 4.2. Structural–Magnetic Combined Analysis

The same steel pipe model as in the structural analysis was used for the static magnetic field analysis. The element type was changed to SOLID226 to calculate the magnetic field inside and outside the pipe model. The pipe model is in a demagnetized state and does not consider magnetostriction and remanent magnetization in the pipe. 

The governing equation used in the structural–magnetic combined analysis is expressed by the following equation:

(28) $$\left[\begin{array}{cc} \left[M\right] & \left[0\right]\\ \left[0\right] & \left[0\right] \end{array}\right]\left\{\begin{array}{c} \left\{\ddot{u}\right\}\\ \left\{\ddot{A}\right\} \end{array}\right\}+\left[\begin{array}{cc} \left[C\right] & \left[0\right]\\ \left[0\right] & \left[0\right] \end{array}\right]\left\{\begin{array}{c} \left\{\dot{u}\right\}\\ \left\{\dot{A}\right\} \end{array}\right\}+\left[\begin{array}{cc} \left[K\right]+\left[K^{uu}\right] & \left[K^{uA}\right]\\ {\left[K^{uA}\right]}^{T} & \left[K^{AA}\right] \end{array}\right]\left\{\begin{array}{c} \left\{u\right\}\\ \left\{A\right\} \end{array}\right\}=\left\{\begin{array}{c} \left\{F\right\}\\ \left\{\psi_{i}\right\} \end{array}\right\},$$

where $\left\{A\right\}$ is the magnetic vector potential, $\left[C\right]$ is the damping matrix, $\left[K^{uu}\right]$ is the stiffness matrix defined from $\left\{u\right\}$ and Maxwell stress, $\left[K^{uA}\right]$ is the stiffness matrix defined from $\left\{A\right\}$ and Maxwell stress, $\left[K^{AA}\right]$ is the magnetic resistivity matrix, {F} is the load vector, and $\left\{\psi_{i}\right\}$ is the coercive force vector. In this simulation, $\left\{\psi_{i}\right\}$ is zero because the demagnetized state is simulated. 

The load was applied at one center position (z = 50 cm) on the outside of the pipe and set to 50,000 Pa in the negative y-axis direction. The damping ratio was set at 0.1%. The boundary conditions for vibration were fixed at both ends of the piping as in the modal analysis. The magnetic resistivity is determined by the relative permeability. The permeability of the pipe and the air were set to 1000 and 1, respectively. A magnetic field was generated in infinite space around the pipe, and a 50 μT DC magnetic field was applied perpendicularly to the length of the steel pipe. 

**Figure 10:**
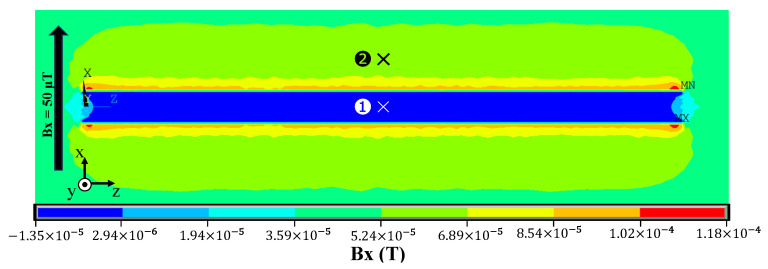
Results of the static-field analysis of the pipe without deformation. The amount of magnetic flux density (*B*_x_) in the cross-section of the x–z plane in the axial direction of the piping was calculated.

The results of the static-field analysis of the pipe without deformation are shown in Figure 10, which shows the longitudinal cross-section of the pipe. As predicted by theory, the geomagnetic field generates leakage fields around the pipe that cause the magnetic field inside the pipe to decay. The magnetic field inside the pipe is given as $B_{in}$, and that outside the pipe is given as $B_{out}$. As shown in Figure 10, the x component of the static magnetic field was calculated for the inner node [labeled (1)] and the outer node [(2)] symmetrical to the piping plane. Figure 10 shows that $B_{in}=0.563\;\mu T$ and $B_{out}=63.7\;\mu T$. The magnetic field inside the pipe is lower than that outside, and the fact that $B_{out}$ is slightly larger than the applied magnetic field is due to the magnetic field generated by the pipe. From these values, the shielding factor is expressed as the ratio of the magnetic field outside and inside the magnetic material obtained as follows:

(29) $$\lambda_{r}=\frac{B_{in}}{B_{out}}.$$

From Equation (29), $\lambda_{r}=0.0088$ for the model as shown in Figure 10. From Equation (17), $\lambda_{r}=0.0084$, which is in good agreement with the theoretical and simulated values. It is therefore concluded that the cause of the attenuation of the magnetic field inside the pipe was the shielding effect, which could be reproduced by Equation (29).

**Figure 11:**
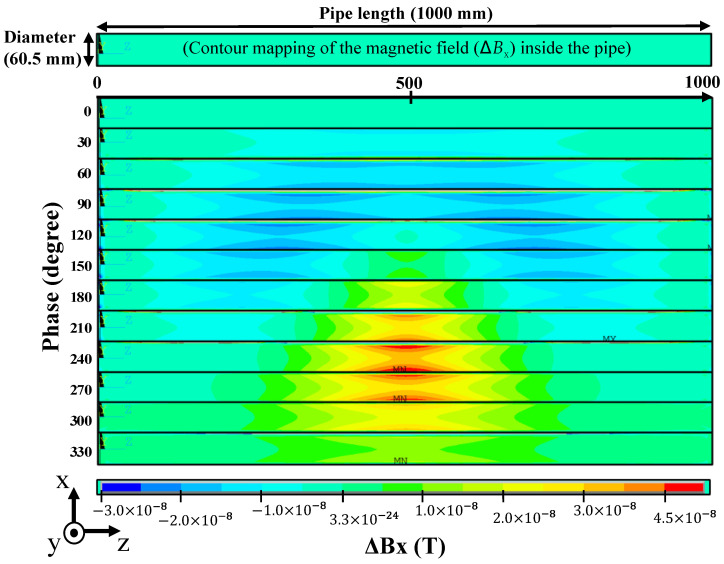
Results of static-field analysis of pipe with deformation. The amount of change in magnetic flux density (Δ*B*_x_) for one period (12 datasets) in a cross-section in the x–z plane was calculated.

The MHT signal is generated by the fluctuation in the magnetic field due to natural oscillations of the pipe, which can be calculated with a continuous static magnetic field analysis [18]. The results of the static-field analysis of the pipe deformed by the natural vibration are shown in Figure 11. The deformation of the pipe model was calculated by frequency-response analysis. In this analysis, mode (n, m) = (2, 1) was chosen, and a cyclic load was applied to make the model resonate. Deformation of the pipe was calculated at phase-angle intervals of 30 degrees, creating a total of 12 datasets for one period of the natural vibration.

The magnetic flux density distribution around the pipe was calculated by applying an external magnetic field in the x-axis direction to 12 datasets obtained by frequency-response analysis. However, the magnetic field inside the pipe is very small compared to that on the outside, so the amount of change in the magnetic field was calculated. To obtain the amount of change in magnetic flux density, the difference between the phase angle of 0° (before deformation) and that of 30° to 330° (after deformation) was calculated.

The x component of the magnetic flux density variation inside the pipe is also shown in Figure 11. Δ*B*_x_ contours were plotted on the cross-section of the piping and aligned every 30 degrees of phase. In mode (n, m) = (2, 1), the largest pipe deformation occurs at the center position (z = 50 cm), and the magnetic field change is also large. The magnetic field oscillated in the pipe is in the order of nT, and Δ*B*_x_ is 21 nT in the x direction and zero in the y and z directions. It is therefore concluded that the static magnetic field in the pipe is damped by the shielding effect, so the damped magnetic field was oscillated by the natural vibration.

### 4.3. Discussion of the Simulation Results

The above simulations were performed to confirm the validity of the experimental results. First, the static magnetic field analysis confirmed that the magnetic field is attenuated by the magnetic shielding effect of the pipe. The simulation results support the experimental results that the magnetic field observed by the sensor is much smaller than the geomagnetic field. Next, the oscillation of the magnetic field due to the natural vibration of the pipe is confirmed by a continuous static magnetic field analysis. This result supports the experimental result that the frequency of the magnetic field is consistent with the natural frequency of the piping. These simulation results corresponded to the experimental results, thus confirming their validity.

However, in terms of the amplitude of vibration, the difference between the experimental result (617 pT) and the simulation result (21 nT) occurred. The reason for this is the difference between the direction of the sensor in the experiment and the component of the magnetic field calculated in the simulation. In the experimental setup, the sensor was oriented according to the axial direction of the pipe. The TMR sensor detects the magnetic field on the axis of sensitivity, but the T-shaped MFCs allow it to also detect a small amount of magnetic field perpendicular to the axis of sensitivity.

The size and orientation of the TMR sensor must be a concern for real-world use of MHT. To inspect piping regardless of its size and orientation, the sensor must be small. According to the structural–magnetic combined analysis, a large magnetic field can be detected when the TMR sensor is oriented in the geomagnetic direction. High sensitivity and low noise are necessary to observe sub-nT magnetic fields with high SNR with a TMR sensor. A larger MFC in the sensor can output a larger signal with a smaller magnetic field. In addition, increasing the number of MTJ devices in series will reduce the noise. However, both of these methods increase the size of the sensor and require the sensor to be oriented in accordance with the axial direction in the pipe. Therefore, if the sensor can be miniaturized and detected by three-dimensional sensing for the magnetic field, piping can be inspected with high sensitivity without having to align the sensor with the geomagnetic field. To expand the utility of MHT in the future, it will be essential to develop a 3-axis TMR sensor with both high sensitivity and low noise.

## 5. Conclusions

This study is the first step in a research effort to apply MHT, which utilizes a very sensitive magnetic sensor, a TMR sensor, for piping inspection. To inspect long-distance pipelines, it is necessary to accommodate future unmanned and manpower-saving inspections. Unmanned and manpower-saving inspection of long-distance pipelines requires robots that inspect for long periods of time, so sensor power consumption must be reduced. Furthermore, noncontact, high-speed, high-precision inspection is required. These requirements can be easily achieved by applying a low-power, high-sensitivity TMR sensor.

The vibration characteristics of steel pipe specimens were investigated experimentally and by structural analysis. A simple experiment on the natural vibration of a pipe using a TMR sensor and a hammer showed that it is possible to measure the thickness of pipes with a very high resolution of several tens of microns, even with no contact between the sensor and pipe wall. The same experiments conducted with underwater pipes housing a waterproofed TMR sensor confirmed the underwater measurement with a minimum resolution of less than 100 μm. These results indicate that the high sensitivity defined by the natural frequency of the piping (about 1000 Hz/mm) and the very low detectivity of the TMR sensor (sub-pT level) enable high-precision detection of slight changes in the shape of the pipes. 

The magnetic properties of the pipes were investigated by using FEM analysis. The results of the structural–magnetic combined analysis demonstrated that the geomagnetic field is shielded by the piping and becomes weak and that the magnetic field oscillates due to the natural vibration of the pipe. The simulation results correspond well with the experimental results and confirm their validity.

These results demonstrate that a TMR sensor with low power consumption (less than 25 mW) allows for noncontact inspection of the pipeline with a short measurement time (5 s) and a high wall thickness resolution (much less than millimeters). These key findings contribute greatly to unmanned and manpower-saving nondestructive inspection of long pipes.

## Figures and Tables

**Table 1:** Analytical results for sensitivity, accuracy, and resolution.

**Thickness (mm)**			5.66 ± 0.1	5.07 ± 0.1	4.82 ± 0.1	4.57 ± 0.1
**Amount of thinning (mm)**			-----	0.50	0.75	1.00
**Above ground**	**Frequency (Hz)**		4776.62 ± 0.04	4437.32 ± 0.09	4061.46 ± 0.33	3665.17 ± 0.03
	**Accuracy (Hz)**		4.39 ± 0.12	4.67 ± 0.24	12.69 ± 1.19	14.17 ± 0.11
	**4 pipes**	**Sensitivity (Hz/mm)**	990.3 ± 155.6			
		**Resolution (μm)**	4.4 ± 0.7	4.7 ± 0.8	12.8 ± 2.3	14.3 ± 2.3
	**3 pipes**	**Sensitivity (Hz/mm)**	-----	1544.3 ± 16.7		
		**Resolution (μm)**	-----	3.0 ± 0.2	8.2 ± 0.8	9.1 ± 0.1
**Underwater**	**Frequency (Hz)**		4120.16 ± 0.08	3579.45 ± 0.30	3443.34 ± 0.14	2989.00 ± 0.26
	**Accuracy (Hz)**		23.22 ± 0.23	32.12 ± 0.90	22.53 ± 0.44	66.43 ± 0.56
	**4 pipes**	**Sensitivity (Hz/mm)**	979.86 ± 108.6			
		**Resolution (μm)**	23.6 ± 2.6	32.8 ± 3.7	23.0 ± 2.6	67.8 ± 7.5
	**3 pipes**	**Sensitivity (Hz/mm)**	-----	1180.9 ± 259.8		
		**Resolution (μm)**	-----	27.2 ± 6.0	19.1 ± 4.2	56.3 ± 12.4

**Table 2:** Setting parameters used in FEM analysis.

Parameters	Steel Pipe	Parameters	Inner Air	Parameters	Outer Air
Diameter (mm)	60.5	Diameter (mm)	54.84	X (mm)	2000
Thickness (mm)	5.66	-----	-----	Y (mm)	2000
Length (mm)	1000	Length (mm)	1000	Z (mm)	3000
Element shape	Hexahedral	Element shape	Hexahedral	Element shape	Tetrahedral
Element size (mm)	10	Element size (mm)	10	Element size (mm)	50
Element type	SOLID186	Element type	SOLID186	Element type	SOLID186
Young’s modulus (Pa)	$2.05\times 10^{11}$	Young’s modulus (Pa)	100	Young’s modulus (Pa)	100
Poisson’s ratio	0.3	Poisson’s ratio	0	Poisson’s ratio	0
Density (kg/m^3^)	7850	Density (kg/m^3^)	1.16	Density (kg/m^3^)	1.16
Relative permeability	1000	Relative permeability	1	Relative permeability	1

## Data Availability

Data are available from the corresponding author upon request.
